# Supplementary material for: The C-terminal tail of polycystin-1 suppresses cystic disease in a mitochondrial enzyme-dependent fashion
Source: Nat Commun. 2023 Mar 30;14:1790. doi: 10.1038/s41467-023-37449-1 (PMC10063565; doi:10.1038/s41467-023-37449-1)
Supplement: Supplementary file 1 — Supplementary Information [file 41467_2023_37449_MOESM1_ESM.pdf]

SUPPLEMENTARY MATERIAL

Supplementary Figure 1

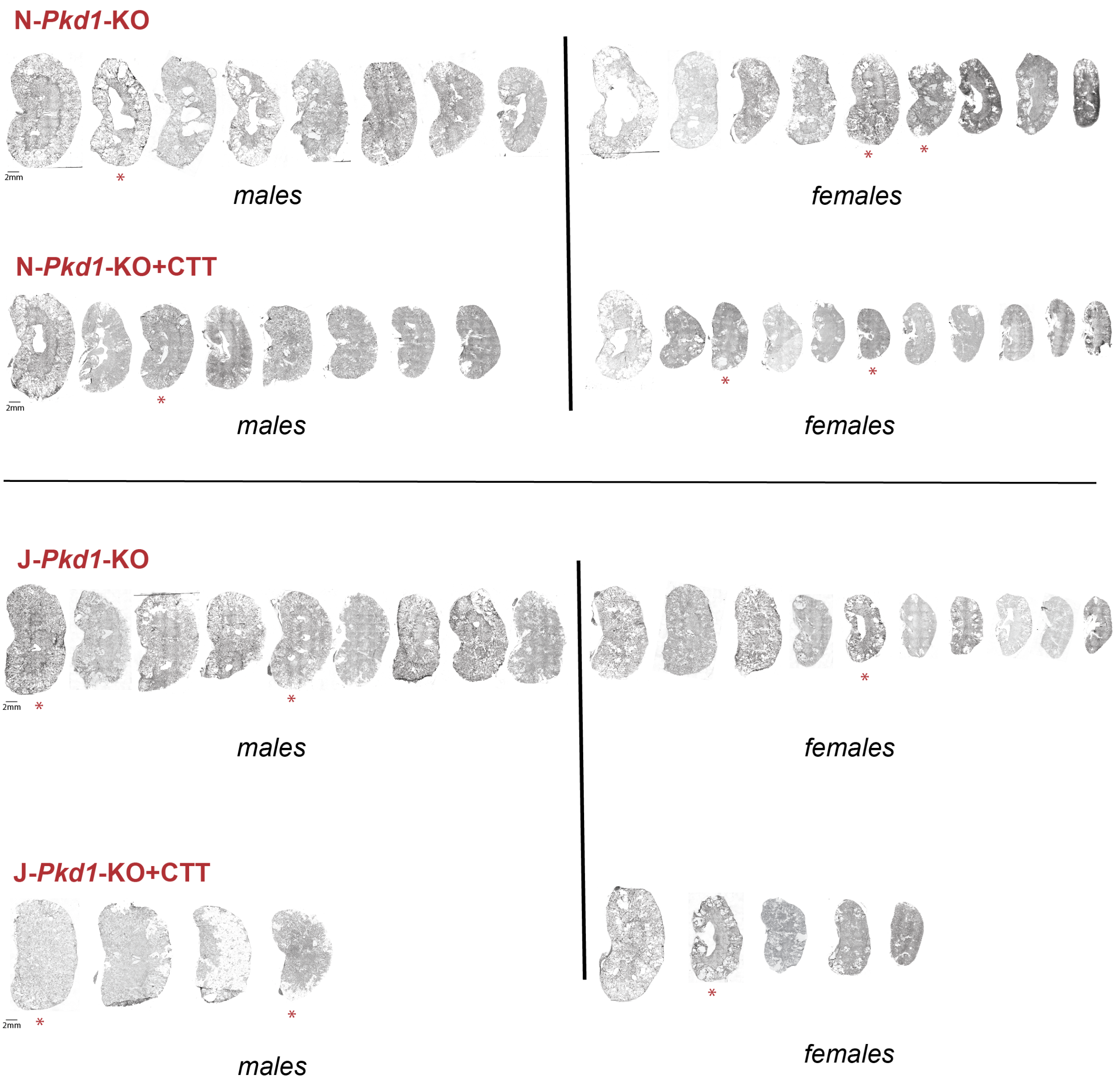

Supplementary Figure 1: Complete image set generated in the evaluation of *Pkd1$^{fl/fl}$;Pax8^{rtTA};TetO-Cre* models (Fig. 1b-e and Fig. 3a-e).

H&E-stained kidney sections (4X) from the four indicated genotypes, separated by sex. Oral doxycycline was administered from weeks 4-6 and all mice were

sacrificed at 16 weeks. The asterisks mark the images used as representative images in Fig. 1e and Fig. 3e. Scale bar: 2mm.

Supplementary Figure 2

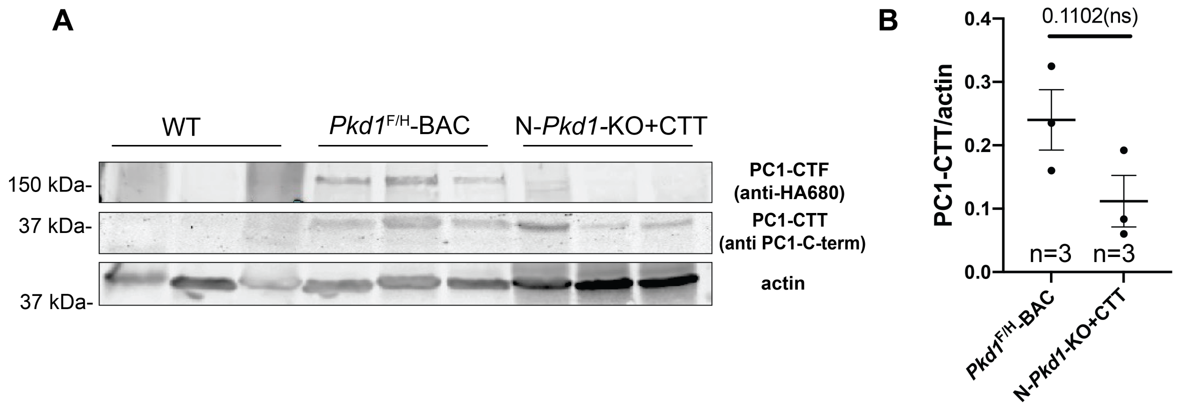

Supplementary Figure 2: Levels of CTT protein detected in N-*Pkd1*-KO+CTT mice are comparable to those detected in *Pkd1*$^{F/H}$-BAC mice

(A-B) Full-length WT PC1 is a low-abundance protein that is not reliably detectable in mouse kidneys[1,2], and its endogenous cleavage products are even less abundant and harder to detect. Thus, to quantitatively analyze the level of CTT produced from cleavage of full-length PC1 protein *in vivo*, we used offspring of a founder of the *Pkd1*$^{F/H}$-BAC line (Tg248) that carries 3 copies of the BAC-*Pkd1* transgene and expresses a PC1 protein with a 3XFlag tag at its N terminus and a 3XHA tag at its C terminus[2,3]. Quantitative western blotting previously determined that Tg248 mice exhibit a 3-fold increase in tagged PC1 expression relative to offspring of a single-copy founder (Tg14)[2]. Since expression of tagged PC1 in the Tg248 line is driven by the endogenous *Pkd1* promoter, it is likely that the 3 copies of the *Pkd1*$^{F/H}$-BAC transgene drive expression of the tagged protein that is roughly comparable to 1.5X the quantity of the native PC1 generated from the 2 native copies of *Pkd1* encoded in the mouse genome. We performed immunoblotting of 60 µg of total kidney lysate from WT, N-*Pkd1*-KO+CTT, and BAC-*Pkd1* mice (A). Actin served as loading control. The 150-kDa bands exclusive to BAC-*Pkd1* mice represent the PC1-

CTF fragment that results from N-terminal cleavage of full-length PC1 at the GPS site[4]. Lysates from *Pkd1*$^{F/H}$-BAC mice showed the same 37-kDa C-terminal HA-tagged tail fragment band as the CTT-expressing *Pkd1*-KO mice (A), which is detected in similar quantities (B), suggesting an upper threshold for CTT expression in the N-*Pkd1*-KO+CTT mice of approximately 1.5-fold above the levels expected for WT mice. Data are expressed as mean ± SEM. Pairwise comparisons were performed using two-tailed Student's t-test. Source data are provided as a Source Data file.

Supplementary Figure 3

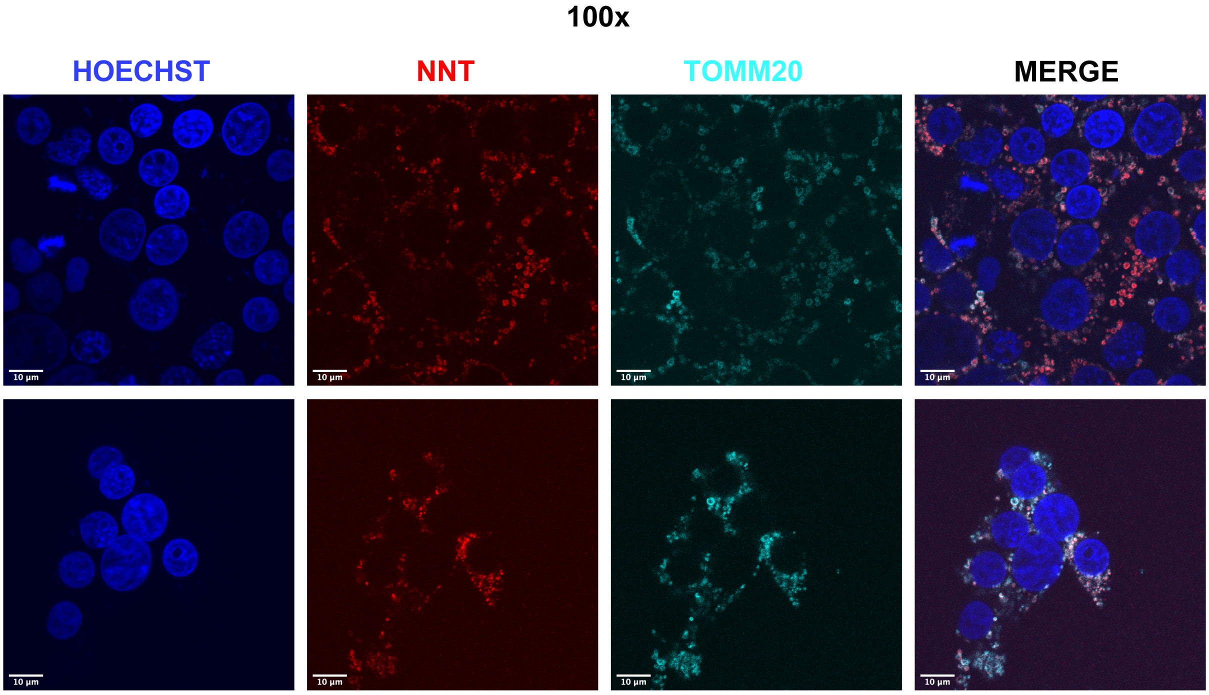

Supplementary Figure 3: Images used in the assessment of NNT and TOMM20 mitochondrial colocalization (related to Fig. 2f)

Representative immunofluorescence (100X) images showing mitochondrial colocalization of endogenous NNT and TOMM20 in HEK293 cells. Mander's colocalization analysis of 23 individual cells obtained from 9 independent images gathered from 3 biological replicates revealed an overlap coefficient of 0.9322 (Fig. 2f). Scale bar: 10μm.

Supplementary Figure 4

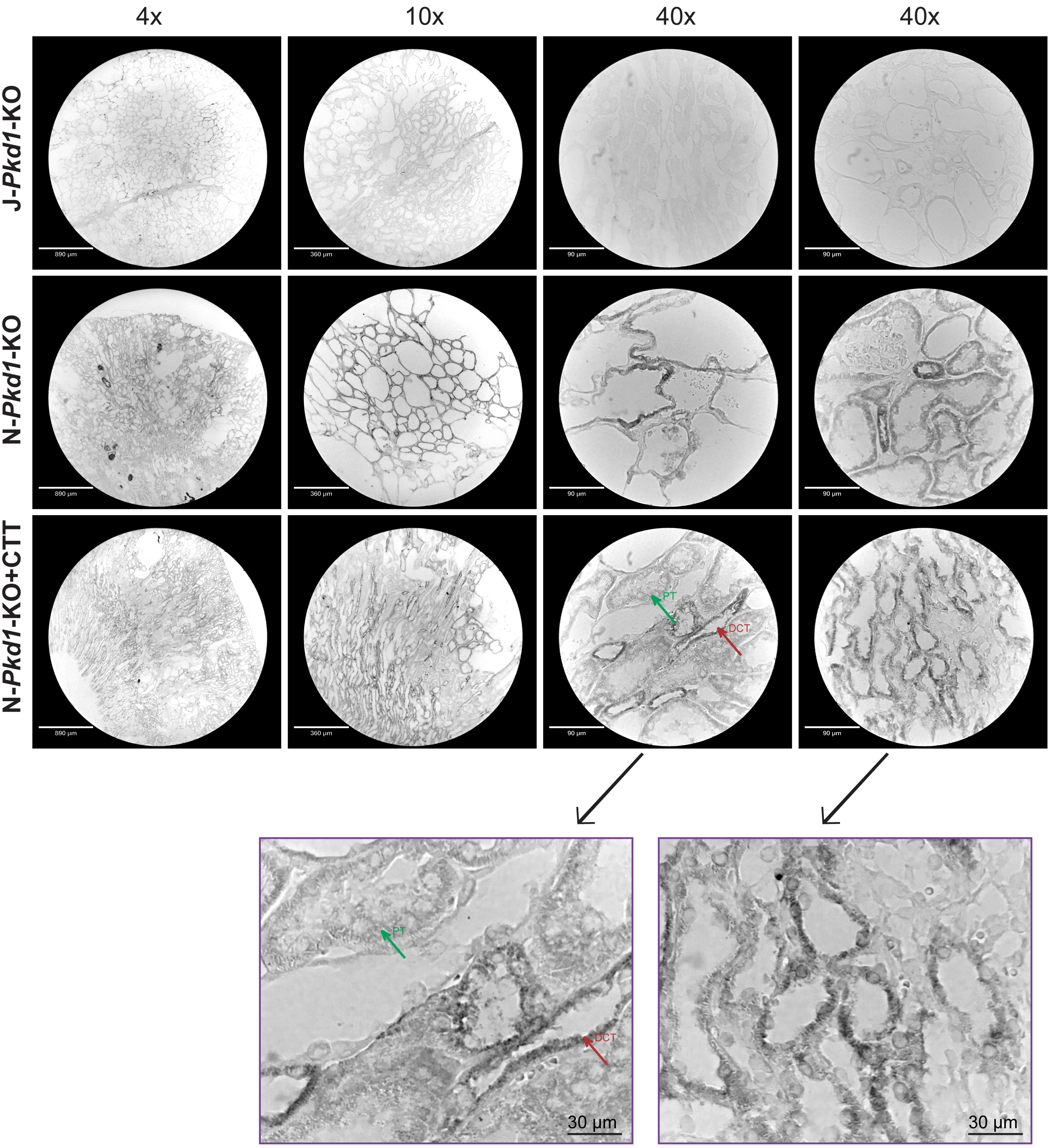

Supplementary Figure 4: NNT expression in mouse renal tissue

Immunohistochemical (IHC) detection of NNT protein expression in kidney sections of J-*Pkd1*-KO, N-*Pkd1*-KO and N-*Pkd1*-KO+CTT mice acquired by transmission light microscopy and processed with ImageJ automatic settings (32-bit images). As expected, these images demonstrate an absence of NNT expression on the "J" background (magnification= 4X, 10X, and 40X, as indicated in figure). Images demonstrate the preferential localization of NNT to distal convoluted tubules (DCT, red) and medullary tubules as compared to proximal tubules (PT, green). No NNT was detected in glomeruli or Bowman's capsule. Enlarged regions of 40X images (purple boxes) demonstrate that the staining pattern is consistent with mitochondrial localization. These images are representative of three independent mouse kidneys per genotype. Three images were obtained per kidney at the 4X and 10X magnifications; 6 images were obtained per kidney at the 40X magnification. Scale bar: 890µm, 360 µm, 90µm, and 30 µm, as indicated in figure.

Supplementary Figure 5

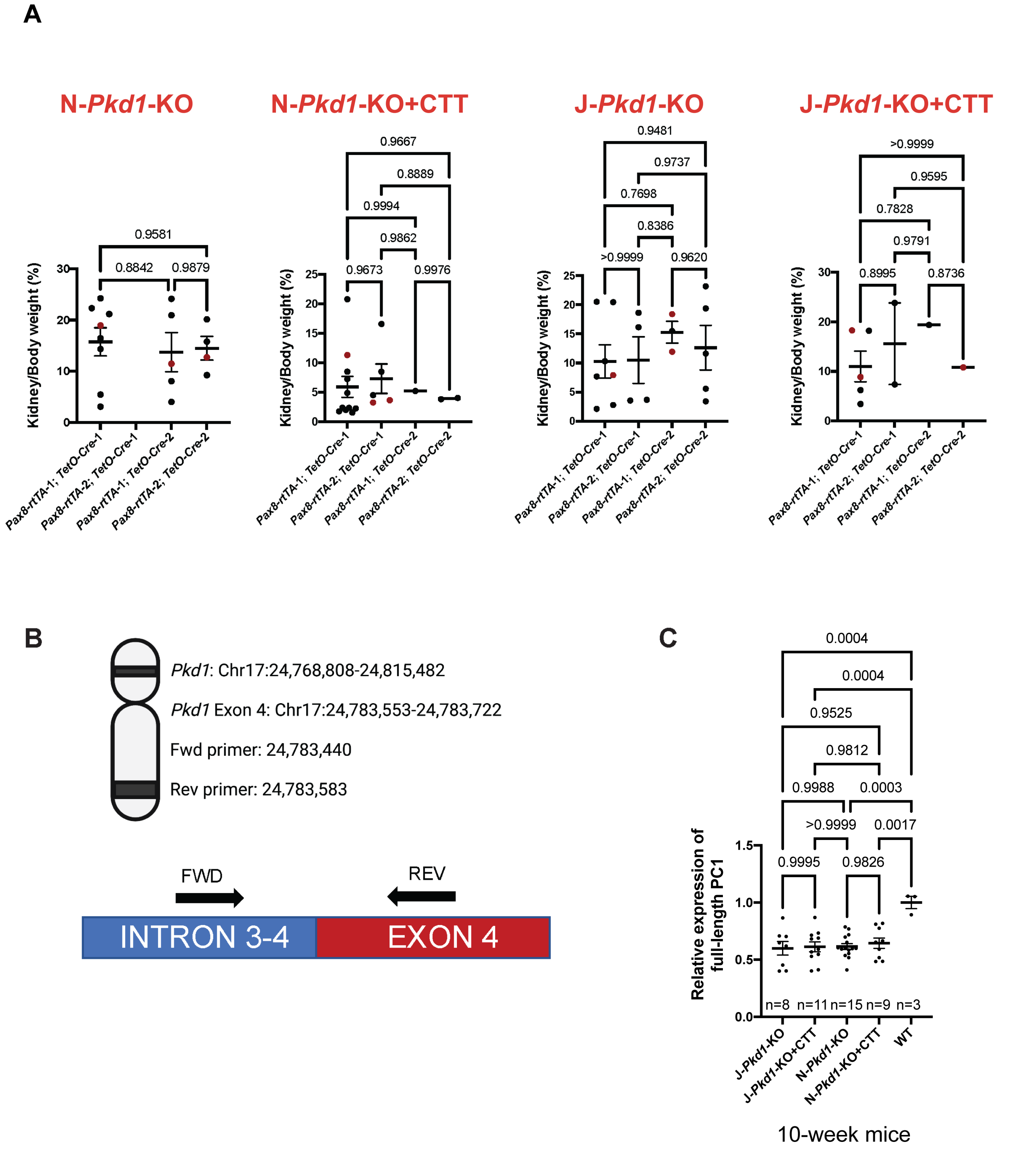

Supplementary Figure 5: *Pax8^{rtTA}* and *TetO-Cre* copy numbers do not correlate with disease severity in the 16-week mouse cohorts, and PC1 rearrangement levels secondary to Cre-mediated recombination are similar across pre-cystic mouse cohorts, prior to the onset of structural changes

(A) *Pkd1*-KO+CTT and *Pkd1*-KO on the "N" and "J" backgrounds show random distribution of homozygosity or heterozygosity status for both *Pax8^{rtTA}* and *TetO-Cre* alleles. These parameters do not correlate with phenotype severity in any of the four groups, as determined by the KW/BW ratio. The mice included in this analysis correspond to the mice shown in Fig. 1b-d and Fig. 3 a-c. Red dots represent the animals shown in Fig. 1e and Fig. 3e.

(B) In the *Pkd1$^{fl/fl}$;Pax8^{rtTA};TetO-Cre* model, oral doxycycline induces activation of *TetO-Cre* under the control of the *Pax8^{rtTA}* promoter, leading to excision of the floxed exon 2-4 region and consequent inactivation of *Pkd1*[5,6]. We show a schematic representation of qPCR primers capable of exclusively detecting genomic DNA sequence encoding full-length endogenous PC1 from cells that did not undergo Cre-recombination in *Pkd1*-KO mice. The reverse primer is specific for *Pkd1* exon 4 and the forward primer is specific to its preceding intron. Primer positions were based on the mouse genome assembly GRCm39.

(C) We determined levels of non-rearranged WT *Pkd1* by extracting genomic DNA from kidney tissue from each mouse contained in the indicated cohorts followed by quantitative genomic PCR using primers described in (B). The levels of non-rearranged WT *Pkd1* were normalized to levels detected in WT controls. The fractional extent of rearrangement is about 40% in total kidney tissue and remains unchanged across the four groups. The upper threshold for experimental variability within WT mice is approximately 15% (shown in figure).

Of note, cohorts of 10-week *Pkd1*-KO mice +/- CTT, which do not manifest the development of cystic phenotype, were employed in this analysis of rearrangement levels. The pre-cystic mouse cohorts are composed of 54%-62% female and 38%-46% male mice.

Data are expressed as mean ± SEM. Multiple group comparisons were performed using one-way ANOVA followed by Tukey's multiple-comparisons test. Source data are provided as a Source Data file.

Supplementary Figure 6

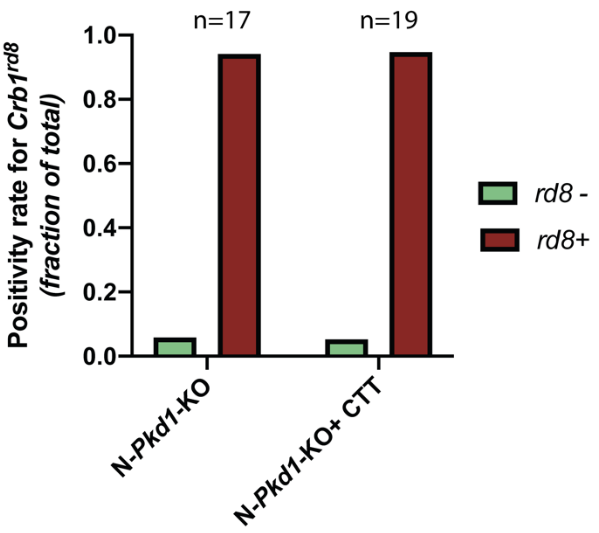

Supplementary Figure 6: Frequency of the *Crb1*^{rd8} mutant allele in cystic mice on the "N" background

The frequency of the *rd8* mutant allele, associated with the C57BL/6N background, was similar in N-*Pkd1*-KO+CTT and N-*Pkd1*-KO mice, as determined by standard genotyping[7] and did not correlate with disease severity. Source data are provided as a Source Data file.

Supplementary Figure 7

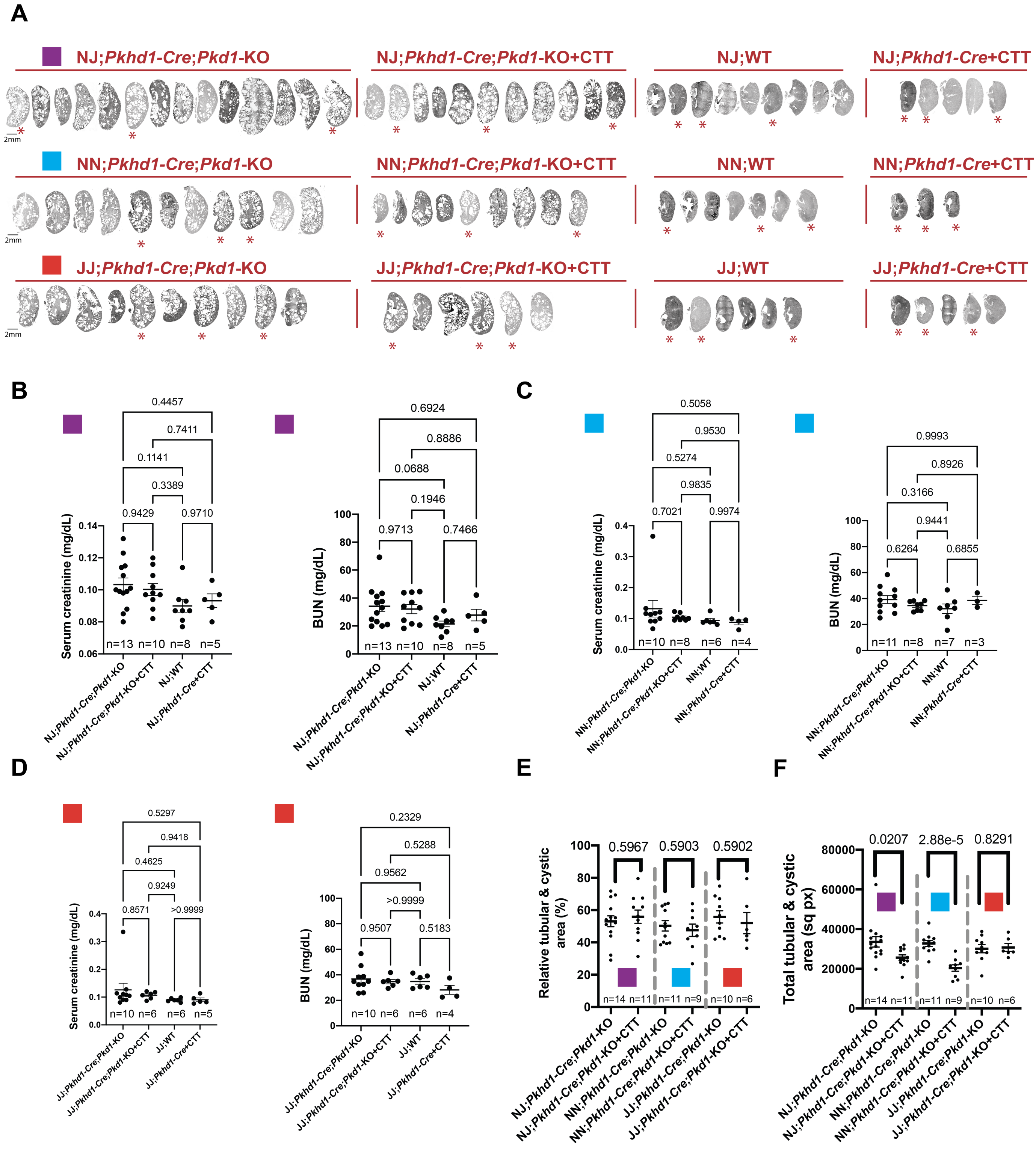

Supplementary Figure 7: Complete image set generated in the evaluation of *Pkd1$^{fl/fl}$Pkhd1-Cre* models (Fig. 4b-g) and characterization of renal function and tubular and cystic area observed in this mouse model.

(A) H&E-stained kidney sections (4X) from NJ F1 (purple box), NN F2 (blue box), and JJ F2 (red box) mice presenting the four indicated genotypes. All mice were sacrificed at p14. The asterisks mark the images used as representative images in Fig. 4e-g. Scale bar: 2mm.

(B-D) Comparative analysis of *Pkhd1-Cre;Pkd1*-KO +/- CTT, *Pkhd1-Cre*+CTT and WT mice from NJ F1 (B; purple box), NN F2 (C; blue box), and JJ F2 (D; red box) generations showed no differences in serum creatinine or BUN levels.

(E-F) Quantification of tubular and cystic area in H&E-stained kidney sections from *Pkhd1-Cre;Pkd1*-KO +/- CTT NJ F1 (purple box), NN F2 (blue box), and JJ F2, as determined by ImageJ using renal cross-section images shown in A. No differences were detected in tubular and cystic area normalized to whole kidney area (E) in any of the groups. Total (absolute) tubular and cystic area was significantly reduced in CTT-expressing NN and NJ, while this parameter was unchanged in CTT-expressing JJ mice.

Data are expressed as mean ± SEM. Pairwise comparisons were performed using two-tailed Student's t-test (E and F). Multiple group comparisons were performed using one-way ANOVA followed by Tukey's multiple-comparisons test (B, C, and D). Source data are provided as a Source Data file.

Supplementary Figure 8

A

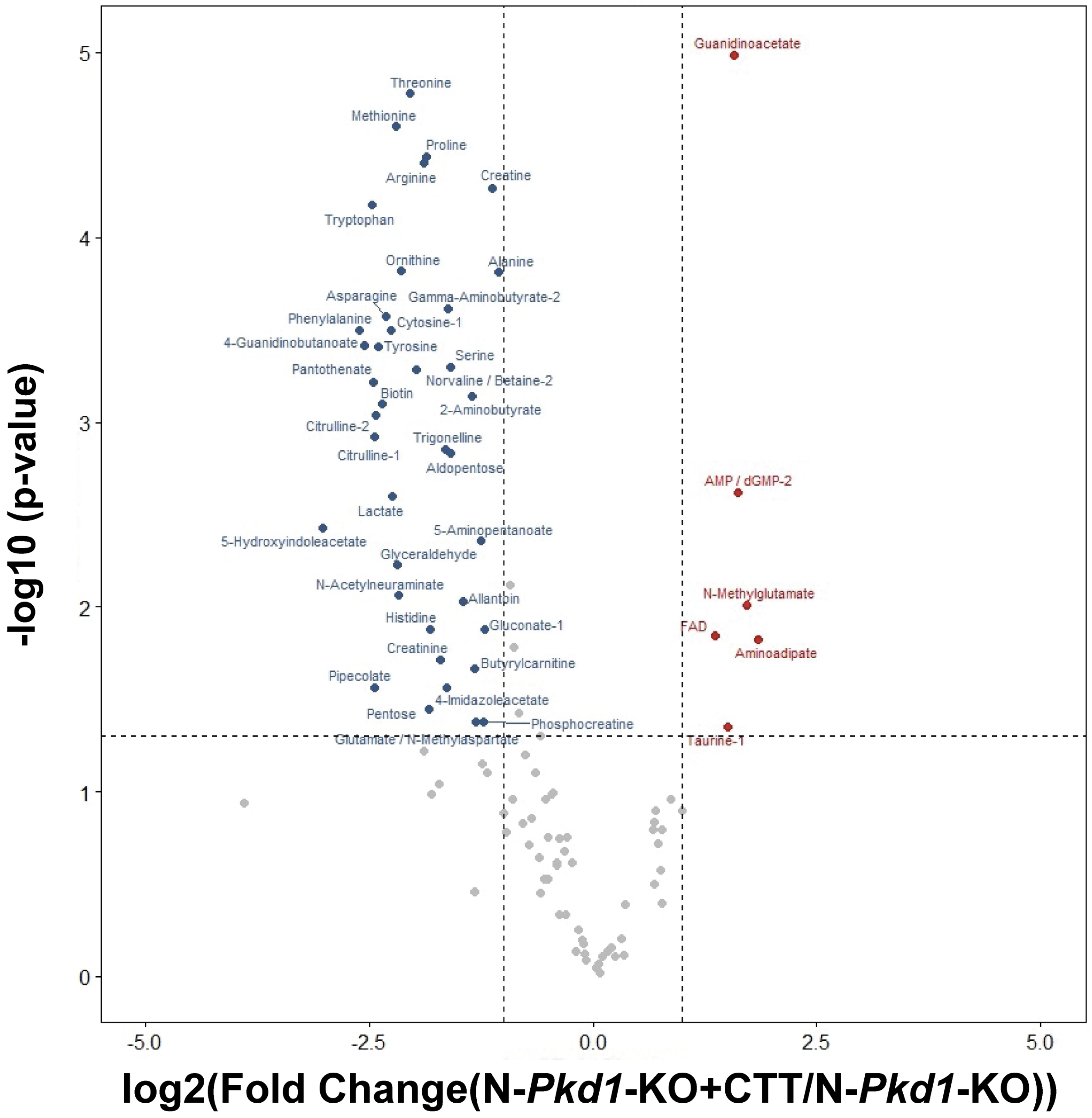

B

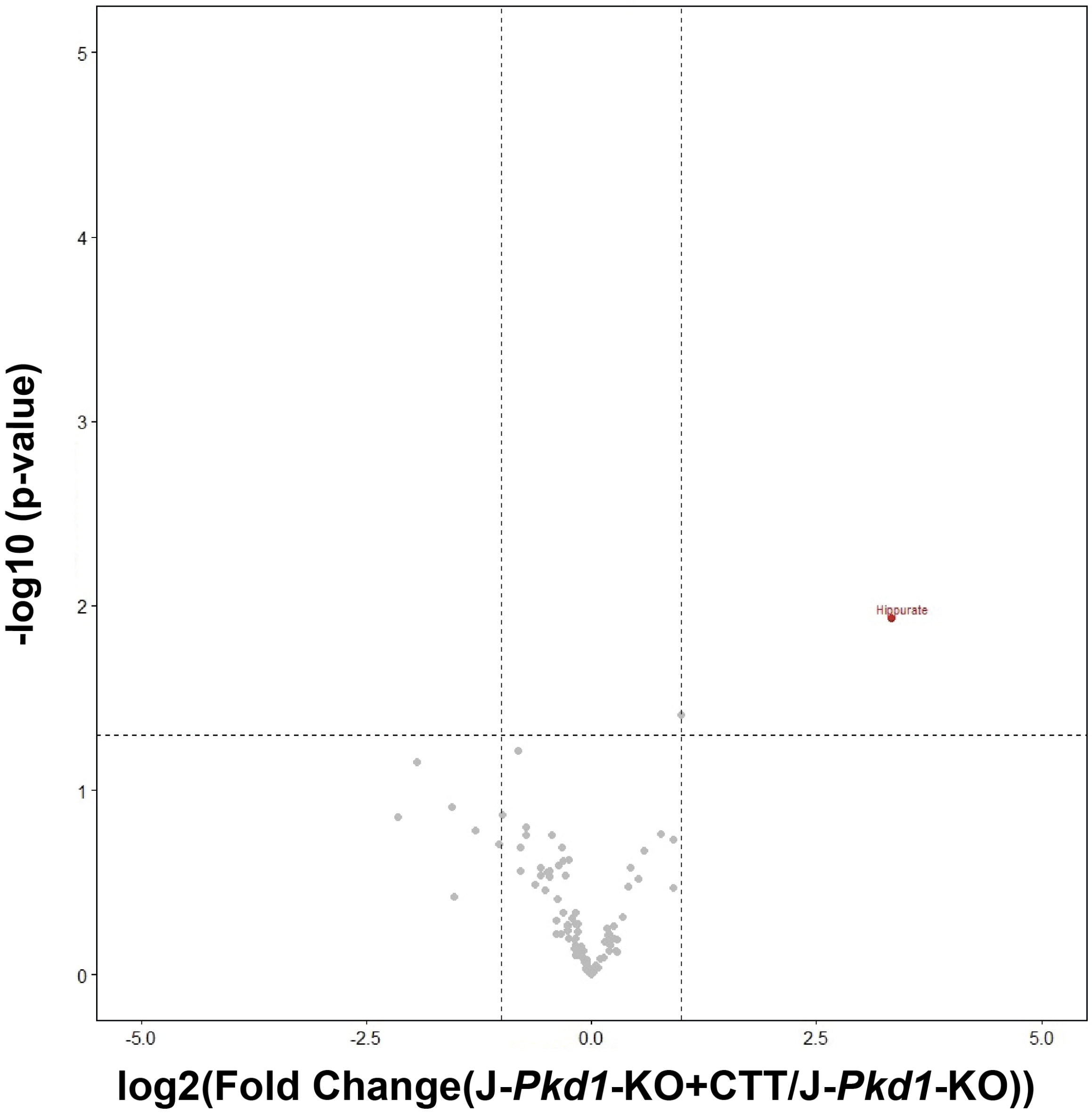

-log10 (p-value)
5
4
3
2
1
0
Hippurate
-5.0
-2.5
0.0
2.5
5.0
log2(Fold Change(J-*Pkd1*-KO+CTT/J-*Pkd1*-KO))

C

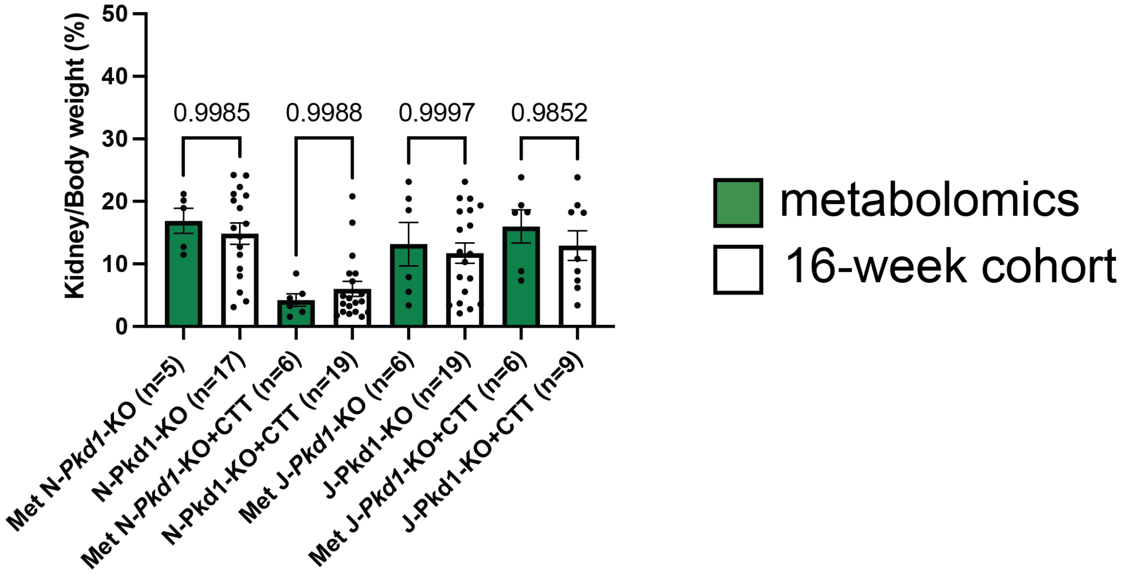

Kidney/Body weight (%)
50
40
30
20
10
0
0.9985
0.9988
0.9997
0.9852
Met N-*Pkd1*-KO (n=5)
N-Pkd1-KO (n=17)
Met N-*Pkd1*-KO+CTT (n=6)
N-Pkd1-KO+CTT (n=19)
Met J-*Pkd1*-KO (n=6)
J-Pkd1-KO (n=19)
Met J-*Pkd1*-KO+CTT (n=6)
J-Pkd1-KO+CTT (n=9)
metabolomics
16-week cohort

Supplementary Figure 8: Differential metabolic profiling of CTT-expressing mice in C57BL/6N and C57BL/6J backgrounds (related to Fig. 5b)

(A-B) Volcano plot identifying each of the metabolites that are differentially detected in kidney extracts from *Pkd1*-KO+CTT mice vs *Pkd1*-KO mice on the "N" (A) and "J" (B) backgrounds (volcano plot shown in Fig. 5b). The vertical lines in each panel mark 2-fold changes; horizontal lines mark $P<0.05$ determined by two-tailed Student's t-test. The set of metabolites that exhibit reduced levels in N-*Pkd1*-KO+CTT mice versus *Pkd1*-KO mice included several metabolites relevant to ADPKD, such as methionine[8],lactate[9,10], asparagine[11,12] and glutamate[11], as well as uremic toxins (allantoin and 5-hydroxyindoleacetate) and urea cycle metabolites. Complete untargeted comparative metabolomic analysis is provided in Supplementary Data 2.

(C)Comparative analysis of KW/BW ratios between the 16-week-old *Pkd1*-KO +/- CTT mice utilized in the characterization of this animal model (Fig. 1b-d and Fig. 3 a-c; white columns) and the 16-week-old *Pkd1*-KO +/- CTT utilized for metabolomic analysis (Fig. 5a,b; green columns). The cystic phenotypes of the animals used in the metabolomic studies are the same as those of the cohort presented in Fig. 1 and Fig. 3.

Data are expressed as mean ± SEM. Multiple group comparisons were performed using one-way ANOVA followed by Tukey's multiple-comparisons test. Source data are provided as a Source Data file.

Supplementary Figure 9

A

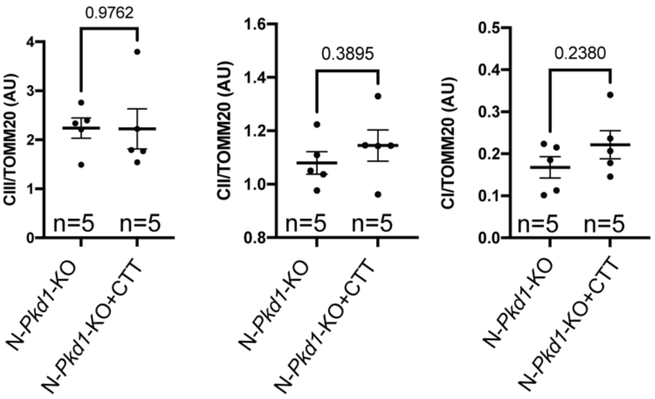

B

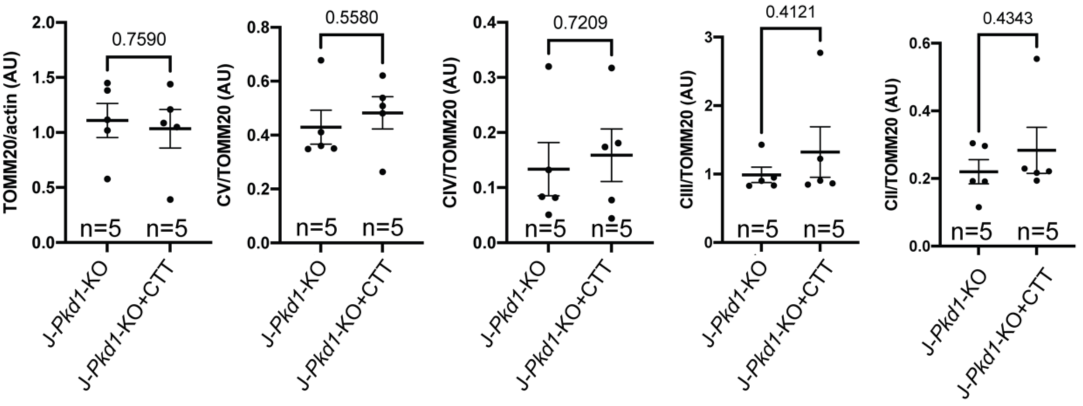

Supplementary Figure 9: Additional comparisons of mitochondrial components in *Pkd1*-KO mice that do or do not express CTT. Relevant to Fig. 5g.

(A-B) Comparison of normalized band intensities representative of both mitochondrial complex assembly and mitochondrial mass for mitochondrial complex components that did not differ between *Pkd1*-KO mice that do or do not express CTT in both "N" (A) and "J" (B) backgrounds.

Data are expressed as mean ± SEM. Pairwise comparisons were performed using two-tailed Student's t-test. Source data are provided as a Source Data file.

Supplementary Figure 10

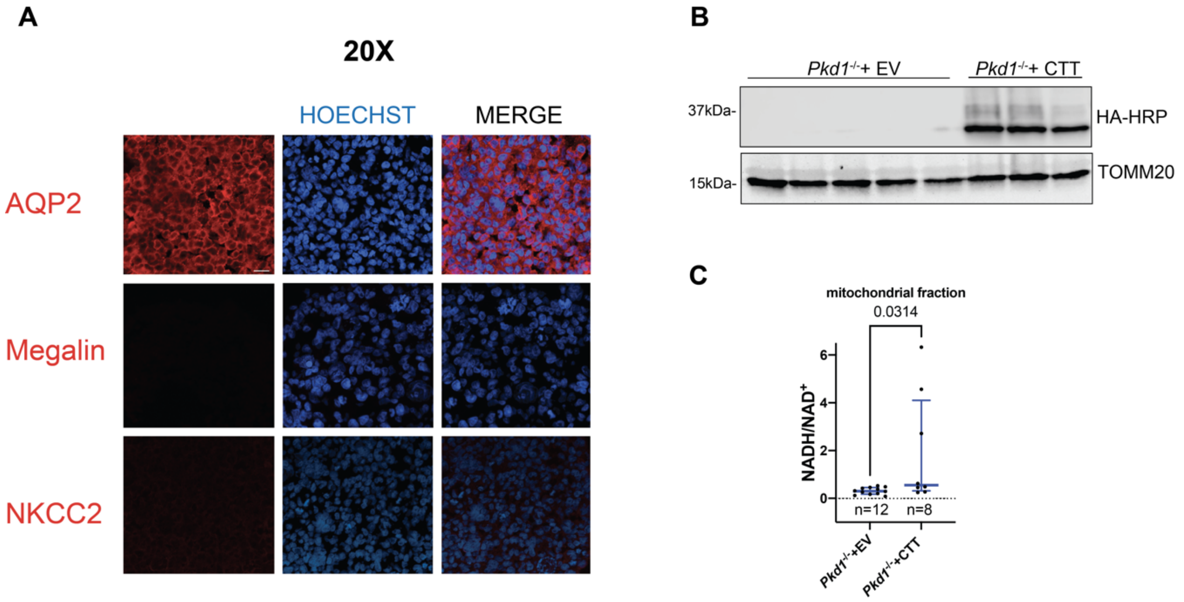

Supplementary Figure 10: Generation of an immortalized *Pkd1*$^{-/-}$ mouse-derived kidney tubule cell line (related to Fig. 7h-j) permits evaluation of CTT-dependent changes in mitochondrial redox

(A) Cells derived from cystic renal tissue obtained from an NN;*Pkhd1-Cre;* Pkd1-KO mouse were transduced with mTERT, selected based on puromycin resistance, and imaged using immunofluorescence microscopy (20X magnification). The selected cell population was comprised predominantly of aquaporin-2 (AQP2) positive collecting-duct cells. Negative staining for megalin (proximal tubule) and NKCC2 (thick ascending limb of the loop of Henle) confirm the identity of the selected cell population. These characterization studies were performed in three independent experiments which produced identical results. Scale Bar: 20μm.

(B) HRP-conjugated anti-HA immunoblotting reveals CTT expression in mitochondrial fractions derived from transfected *Pkd1*$^{-/-}$ cells.

(C) Colorimetric-based measurements of mitochondrial NADH/NAD^+ revealed baseline ratios of ~0.3 in *Pkd1*$^{-/-}$ cells transfected with empty pcDNA3.1 vector (EV), similar to mitochondrial NADH/NAD^+ ratios previously reported in literature (~0.1-0.2)[13]. CTT transfection led to a ~6-fold increase in mitochondrial NADH/NAD^+.

Non-parametric data are depicted with the median and interquartile range (blue bars). Pairwise comparison was performed using two-tailed Mann-Whitney U test. Source data are provided as a Source Data file.

Supplementary Figure 11

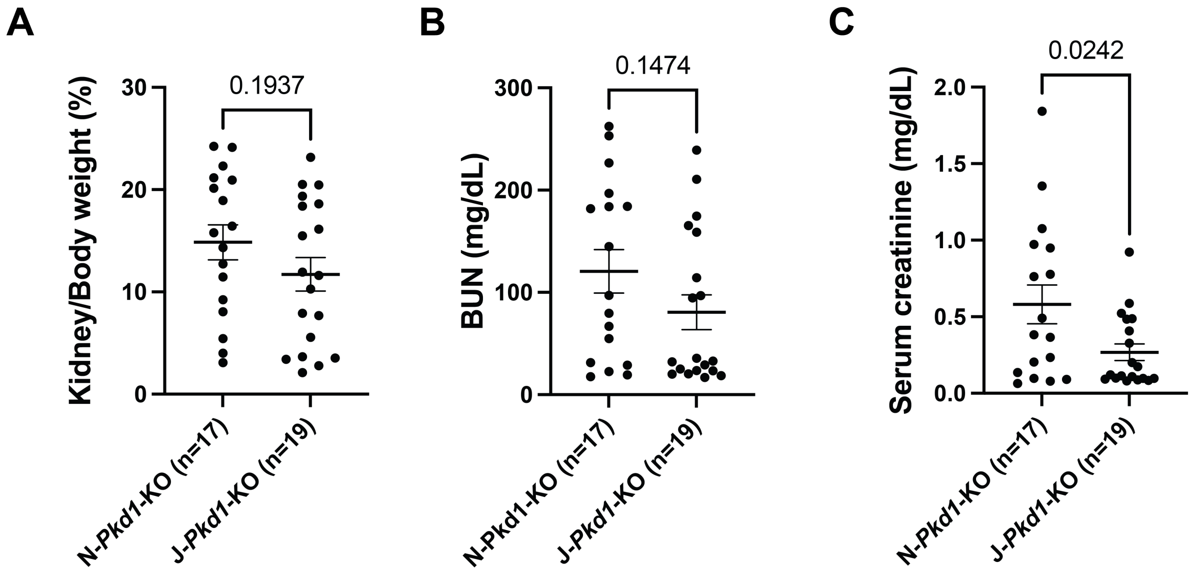

Supplementary Figure 11: Replotting of data from Figures 1 and 3 to examine baseline strain differences between "N" vs "J" *Pkd1*-KO 16-week mouse

(A-C) Comparative analysis of N-*Pkd1*-KO and J-*Pkd1*-KO mice showing the extent of differences in KW/BW ratio (A), BUN (B), and serum creatinine (C). Serum creatinine values show statistically significant differences, and are more elevated in the "N" vs the "J" *Pkd1*-KO mice. Data are expressed as mean ± SEM. Pairwise comparisons were performed using two-tailed Student's t-test. Source data are provided as a Source Data file.

REFERENCES

1. Lin, C.C. *et al.* A cleavage product of Polycystin-1 is a mitochondrial matrix protein that affects mitochondria morphology and function when heterologously expressed. *Sci Rep* **8**, 2743 (2018).
2. Cai, Y. *et al.* Altered trafficking and stability of polycystins underlie polycystic kidney disease. *J Clin Invest* **124**, 5129-44 (2014).
3. Fedeles, S.V. *et al.* A genetic interaction network of five genes for human polycystic kidney and liver diseases defines polycystin-1 as the central determinant of cyst formation. *Nat Genet* **43**, 639-47 (2011).
4. Qian, F. *et al.* Cleavage of polycystin-1 requires the receptor for egg jelly domain and is disrupted by human autosomal-dominant polycystic kidney disease 1-associated mutations. *Proc Natl Acad Sci U S A* **99**, 16981-6 (2002).
5. Shibazaki, S. *et al.* Cyst formation and activation of the extracellular regulated kinase pathway after kidney specific inactivation of Pkd1. *Hum Mol Genet* **17**, 1505-16 (2008).
6. Ma, M., Tian, X., Igarashi, P., Pazour, G.J. & Somlo, S. Loss of cilia suppresses cyst growth in genetic models of autosomal dominant polycystic kidney disease. *Nat Genet* **45**, 1004-12 (2013).
7. Mehalow, A.K. *et al.* CRB1 is essential for external limiting membrane integrity and photoreceptor morphogenesis in the mammalian retina. *Hum Mol Genet* **12**, 2179-89 (2003).
8. Ramalingam, H. *et al.* A methionine-Mettl3-N(6)-methyladenosine axis promotes polycystic kidney disease. *Cell Metab* **33**, 1234-1247 e7 (2021).

9. Rowe, I. *et al.* Defective glucose metabolism in polycystic kidney disease identifies a new therapeutic strategy. *Nat Med* **19**, 488-93 (2013).

10. Chiaravalli, M. *et al.* 2-Deoxy-d-Glucose Ameliorates PKD Progression. *J Am Soc Nephrol* **27**, 1958-69 (2016).

11. Podrini, C. *et al.* Dissection of metabolic reprogramming in polycystic kidney disease reveals coordinated rewiring of bioenergetic pathways. *Commun Biol* **1**, 194 (2018).

12. Baliga, M.M. *et al.* Metabolic profiling in children and young adults with autosomal dominant polycystic kidney disease. *Sci Rep* **11**, 6629 (2021).

13. Yang, Y. & Sauve, A.A. NAD(+) metabolism: Bioenergetics, signaling and manipulation for therapy. *Biochim Biophys Acta* **1864**, 1787-1800 (2016).
