# Supplementary material for: The C-terminal tail of polycystin-1 suppresses cystic disease in a mitochondrial enzyme-dependent fashion
Source: Nat Commun. 2023 Mar 30;14:1790. doi: 10.1038/s41467-023-37449-1 (PMC10063565; doi:10.1038/s41467-023-37449-1)
Supplement: Supplementary file 3 — Description of Additional Supplementary Files [file 41467_2023_37449_MOESM3_ESM.pdf]

Description of Additional Supplementary Files

Supplementary Data 1: Comparative proteomic analysis of material immunoprecipitated from crude renal mitochondrial fractions with anti-HA antibodies from *Pkd1*$^{F/H}$-BAC and WT mice, generated on mixed backgrounds (related to Figure 2C).

The table lists the X values (log2 Fold change BAC/control) and Y values (-log10 *P* value), as depicted in the volcano plot in Figure 2C, for all identified peptides and indicates the targets with *P* value <0.05 determined by two-tailed Fisher's exact test

Supplementary Data 2: Untargeted metabolomic analysis comparing lysates of kidneys from *Pkd1*-KO and *Pkd1*-KO+CTT mice on the "N" and "J" backgrounds, sacrificed at 16 weeks of age (related to Fig. 5a,b and Supplementary Fig. 7).

The table lists metabolites detected by mass spectrometry analysis, as well as the comparative analysis with fold-change and *P* values determined by two-tailed Student's t-test, performed on both backgrounds.

Supplementary Data 3: Untargeted metabolomic analysis comparing lysates of kidneys from *Pkd1*-KO and *Pkd1*-KO+CTT mice on the "N" background, sacrificed at 10 weeks of age (related to Fig. 6 e,f).

The table lists metabolites detected by mass spectrometry analysis, as well as the comparative analysis with fold change and *P* values determined by two-tailed Student's t-test.
