# Supplementary material for: The C-terminal tail of polycystin-1 suppresses cystic disease in a mitochondrial enzyme-dependent fashion
Source: Nat Commun. 2023 Mar 30;14:1790. doi: 10.1038/s41467-023-37449-1 (PMC10063565; doi:10.1038/s41467-023-37449-1)
Supplement: Supplementary file 7 — Reporting Summary [file 41467_2023_37449_MOESM7_ESM.pdf]

nature portfolio

Corresponding author(s): Dr. Michael J. Caplan

Last updated by author(s): Mar 14, 2023

Reporting Summary

Nature Portfolio wishes to improve the reproducibility of the work that we publish. This form provides structure for consistency and transparency in reporting. For further information on Nature Portfolio policies, see our Editorial Policies and the Editorial Policy Checklist.

Statistics

For all statistical analyses, confirm that the following items are present in the figure legend, table legend, main text, or Methods section.

n/a	Confirmed	
☐	☒	The exact sample size (*n*) for each experimental group/condition, given as a discrete number and unit of measurement
☐	☒	A statement on whether measurements were taken from distinct samples or whether the same sample was measured repeatedly
☐	☒	The statistical test(s) used AND whether they are one- or two-sided<br>*Only common tests should be described solely by name; describe more complex techniques in the Methods section.*
☐	☒	A description of all covariates tested
☐	☒	A description of any assumptions or corrections, such as tests of normality and adjustment for multiple comparisons
☐	☒	A full description of the statistical parameters including central tendency (e.g. means) or other basic estimates (e.g. regression coefficient) AND variation (e.g. standard deviation) or associated estimates of uncertainty (e.g. confidence intervals)
☐	☒	For null hypothesis testing, the test statistic (e.g. *F*, *t*, *r*) with confidence intervals, effect sizes, degrees of freedom and *P* value noted<br>*Give P values as exact values whenever suitable.*
☒	☐	For Bayesian analysis, information on the choice of priors and Markov chain Monte Carlo settings
☒	☐	For hierarchical and complex designs, identification of the appropriate level for tests and full reporting of outcomes
☒	☐	Estimates of effect sizes (e.g. Cohen's *d*, Pearson's *r*), indicating how they were calculated

Our web collection on statistics for biologists contains articles on many of the points above.

Software and code

Policy information about availability of computer code

Data collection	All software we use in the manuscript are described in published literature.<br>Confocal images were obtained using a Zeiss LSM780 confocal microscope and the associated software ZEN version 3.6 blue edition (https://www.zeiss.com/microscopy/us/products/microscope-software/zen.html).<br>Whole kidney images from hematoxylin and eosin–stained sagittal kidney sections were obtained at a 4x magnification using automated image acquisition by the scan slide module in the MetaMorph software (Version 7.7, Universal imaging).<br>Immunoblotting was performed using either the Odyssey Infrared Imager and application software version 3.0.25 (Li-Cor Biosciences) or Odyssey Fc and Studio Lite Western Blot Analysis Software version 5.2 (Li-Cor Biosciences).
Data analysis	All software we use in the manuscript are described in published literature.<br>Statistical analyses were performed using GraphPad Prism 9.0.2 (https://www.graphpad.com/scientific-software/prism/).<br>Metabolomic data was analyzed using ClustVis 2.0 (https://biit.cs.ut.ee/clustvis/) and R 4.1.3 (https://www.r-project.org/).<br>Compound Discoverer 3.3 was used to select peaks and integrate intensity from raw metabolomic data (https://www.thermofisher.com/us/en/home/industrial/mass-spectrometry/liquid-chromatography-mass-spectrometry-lc-ms/lc-ms-software/multi-omics-data-analysis/compound-discoverer-software.html).<br>The LC–MS/MS Xcalibur ".raw" acquired proteomic data were processed with Proteome Discoverer 2.0 Thermo Fisher Scientific software and the protein identification was carried out using the Mascot search algorithm (Matrix Science, version 2.6). Proteomic data was analyzed further using Scaffold Proteome Software 5.2.2 (http://www.proteomesoftware.com/products).<br>Xcalibur 3.0 was used to analyze NAD(P)(H) levels in kidney extracts.<br>Cystic index calculation and quantification of individual immunoblot bands was performed using the ImageJ software version 1.52q (https://imagej.nih.gov/ij/, NIH).<br>Region of interest (ROI) in immunofluorescence experiments performed on transfected HEK293 cells were determined using Fiji 3-Image J

software 2.3.0 (NIH) and signal colocalization was assessed using the Coloc 2-ImageJ plugin 3.0.

For manuscripts utilizing custom algorithms or software that are central to the research but not yet described in published literature, software must be made available to editors and reviewers. We strongly encourage code deposition in a community repository (e.g. GitHub). See the Nature Portfolio guidelines for submitting code & software for further information.

Data

Policy information about availability of data

All manuscripts must include a data availability statement. This statement should provide the following information, where applicable:

- Accession codes, unique identifiers, or web links for publicly available datasets
- A description of any restrictions on data availability
- For clinical datasets or third party data, please ensure that the statement adheres to our policy

The mass spectrometry proteomics data have been deposited to the ProteomeXchange Consortium via the PRIDE partner repository under the dataset identifier : accession code PXD040210 [http://www.ebi.ac.uk/pride/archive/projects/PXD040210] (project webpage) and [ftp://ftp.pride.ebi.ac.uk/pride/data/archive/2023/02/PXD040210] (FTP download) . The mass spectrometry metabolomics data have been deposited to the EMBL-EBI MetaboLights database under the dataset identifier: accession code MTBLS7319 [https://www.ebi.ac.uk/metabolights/MTBLS7319] . Source data are provided with this paper. Full-length immunoblots are provided in the Source Data File and Supplementary Information. The following databases/datasets were employed in the planning and analysis of experiments reported in this study: the SWISS-PROT Mus musculus protein database (2017), the mouse genome assembly GRCm39, the MitoCarta 2.0 mitochondrial proteome, the Human Protein Atlas and the Rat Kidney Tubule Expression Atlas.

Human research participants

Policy information about studies involving human research participants and Sex and Gender in Research.

Reporting on sex and gender	Samples were surgically harvested according to the guidelines established by the Institutional Review Board of the University of Maryland and were then de-identified. Sex information was not available.
Population characteristics	Samples were surgically harvested according to the guidelines established by the Institutional Review Board of the University of Maryland and were then de-identified. Population information was not available.
Recruitment	Patients were not recruited for this study.
Ethics oversight	The Baltimore Polycystic Kidney Disease Research and Clinical Core Center (P30DK090868) provided de-identified human kidney tissue used in this study. Samples were surgically harvested according to the guidelines established by the Institutional Review Board of the University of Maryland.

Note that full information on the approval of the study protocol must also be provided in the manuscript.

Field-specific reporting

Please select the one below that is the best fit for your research. If you are not sure, read the appropriate sections before making your selection.

☒ Life sciences ☐ Behavioural & social sciences ☐ Ecological, evolutionary & environmental sciences

For a reference copy of the document with all sections, see nature.com/documents/nr-reporting-summary-flat.pdf

Life sciences study design

All studies must disclose on these points even when the disclosure is negative.

Sample size	Sample sizes for experiments involving Pkd1fl/fl;Pax8rtTA;TetO-Cre mice were chosen based on previous analyses that have examined similar questions with the same experimental animal system (Dong, K. et al. Renal plasticity revealed through reversal of polycystic kidney disease in mice, Nat Genet, 2021 and Ma, M., Tian, X., Igarashi, P., Pazour, G.J. & Somlo, S. Loss of cilia suppresses cyst growth in genetic models of autosomal dominant polycystic kidney disease, Nat Genet 2013).<br>Power calculations were performed prospectively for the F1 Pkd1fl/fl;Pkhd1-Cre (+/- CTT) cohort, based on the CTT-dependent phenotype suppression previously observed in the Pkd1fl/fl;Pax8rtTA;TetO-Cre model and on the observed variation in kidney-to-body weight ratios at p14 in cystic animals without CTT expression, which indicated that 12 animals per group would give 80% power to detect a 35% change in kidney-to-body weight ratio at a significance threshold of P<0.05.<br>For non-animal experiments, sample sizes were chosen based on our previous experience with the relevant assay systems.
Data exclusions	The only mice excluded from the present study were those below the 3rd percentile of body weight derived from the Pkd1fl/fl;Pkhd1-Cre(+-CTT) cohort, sacrificed at p14, to ensure that naturally occurring developmentally delayed runt pups (Burkholder, T et al. Health Evaluation of Experimental Laboratory Mice. Current protocols in mouse biology vol. 2: 145-165, 2012) would not bias the present analyses. There is no correlation between the occurrence of runts and the Pkd1fl/fl;Pkhd1-Cre genotype (Fedeles, S.V. et al. A genetic interaction network of five genes for human polycystic kidney and liver diseases defines polycystin-1 as the central determinant of cyst formation. Nat Genet 43, 639-47,2011; Patel,V et al. Acute kidney injury and aberrant planar cell polarity induce cyst formation in mice lacking renal cilia. Hum Mol

Genet, 17(11):1578-90, 2008; Cai, Y. et al. Altered trafficking and stability of polycystins underlie polycystic kidney disease. J Clin Invest 124, 5129-44, 2014). Five out of the 101 total pups from the NJ F1, NN F2 and JJ F2 cohorts were excluded, one NJ;WT, one NJ;Pkd1fl/fl;Pkhd1-Cre, one NN;Pkd1fl/+;Pkhd1-Cre+CTT, one JJ;WT, and one JJ;Pkd1fl/+;Pkhd1-Cre+CTT.

No other mice or data were excluded from analyses.

Replication

The general outcome of CTT-dependent suppression of cystic disease in NNT-competent strains was replicated in 2 different Pkd1-KO mouse models provided in this publication (2HA-PC1-CTT; Pkd1fl/fl;Pax8rtTA;TetO-Cre and 2HA-PC1-CTT; Pkd1 fl/fl; Pkhd1-Cre). These findings replicate previous in vitro outcomes showing that this same construct decreases cellular proliferation and the cross-sectional area of cysts formed by Pkd1-KO cells in 3D culture (Merrick, D. et al. The gamma-secretase cleavage product of polycystin-1 regulates TCF and CHOP mediated transcriptional activation through a p300-dependent mechanism. Dev Cell 22, 197-210, 2012). Individual replicates are described in figures, figure legends, methods, and supplementary material. All studies successfully replicated the outcomes and there were no studies that failed to replicate the outcome.

Randomization

The allocation of animals in each group was based exclusively on genotype without any exclusion. We ensured that sexually mature adult mice presented similar sex distribution across all experimental groups, as described in figure legends. For all non-animal experiments, in-vitro samples were randomly assigned to the relevant experiment protocols.

Blinding

The investigators were blinded to group allocation during data collection and analysis. For example, serum creatinine and BUN analyses were performed by the George M. O'Brien Kidney Center at Yale University by an individual who was not acquainted with the present study. Quantification of the percentage of ki67 positive nuclei in renal epithelial cells and quantification of cystic index was also performed by individuals blinded to gentype.

Reporting for specific materials, systems and methods

We require information from authors about some types of materials, experimental systems and methods used in many studies. Here, indicate whether each material, system or method listed is relevant to your study. If you are not sure if a list item applies to your research, read the appropriate section before selecting a response.

Materials & experimental systems

n/a	Involved in the study
☐	☒ Antibodies
☐	☒ Eukaryotic cell lines
☒	☐ Palaeontology and archaeology
☐	☒ Animals and other organisms
☒	☐ Clinical data
☒	☐ Dual use research of concern

Methods

n/a	Involved in the study
☒	☐ ChIP-seq
☒	☐ Flow cytometry
☒	☐ MRI-based neuroimaging

Antibodies

Antibodies used

All antibodies we use in the manuscript are described in published literature.
Primary/ Conjugated Primary antibodies
1. Anti-NNT, Invitrogen, Cat# 459170, RRID: AB_2532230. Immunofluorescence (IF) 1:100; immunoblotting (IB) 1:1,000.
2. Anti-NNT(G-8), Santa Cruz, Cat# sc-390215. IB 1:1,000; immunohistochemistry (IHC) 1:50.
3. Anti NNT(B3)-HRP, Santa Cruz, Cat# sc-390236 HRP. IB 1:500.
4. Anti-PC1(C-term), Kerafast, Cat# EJH002. IF 1:100; IB 1:1,000.
5. Anti-HA-Peroxidase (3F10), Roche, Cat# 12013819001; RRID: AB_390917. IB 1:500
6. Anti-HA-680, Thermo Fischer Scientific, Cat# 26183-D680; RRID: AB_2533054. IB 1:500.
7. Anti-ki67, Vector Laboratories, Cat# VP-RM04; RRID: AB_2336545. IF 1:100
8. Anti- α5 subunit Na+K+ ATPase, Developmental Studies Hybridoma Bank, Cat# a5; RRID: AB_2166869. IF 1:100
9. Anti-actin, Sigma, Cat# A2228; RRID: AB_476697. IB 1:1,000.
10. Anti-TOMM20, Novus Biologicals, Cat# NBP1-81556, RRID:AB_11003249. IF 1:100, IB 1:1,000.
11. Anti-Total OXPHOS Cocktail, Abcam, Cat# MS604-300; RRID: AB_1622581. IB 1:1,000
12. Anti-VDAC-HRP, Santa Cruz, Cat# sc-390996 HRP; RRID:AB_2750920. IB 1:250
13. Anti-aquaporin-2 (C-17), Santa Cruz, Cat# sc-9882; RRID:AB_2289903. IF 1:100
14. Anti-NKCC2 - validated in Lytle, C., Xu, J.C., Biemesderfer, D. & Forbush, B., 3rd. Distribution and diversity of Na-K-Cl cotransport proteins: a study with monoclonal antibodies. Am J Physiol 269, C1496-505 (1995). IF 1:100. This antibody is not commercially available.
15. Anti-megalin - validated in Zou, Z., Chung, B., Nguyen, T., Mentone, S., Thomson, B. & Biemesderfer, D. Linking receptor-mediated endocytosis and cell signaling: evidence for regulated intramembrane proteolysis of megalin in proximal tubule. J Biol Chem 279, 34302-10 (2004). IF 1:100. This antibody is not commercially available.

Secondary antibodies (immunoblotting)
1. IRDye® 680RD Goat-anti-Mouse Antibody, Li-Cor, Cat# 926-68070. IB 1:5000.
2. IRDye® 800CW Goat anti-Rabbit IgG Secondary Antibody, Li-Cor, Cat#926-32211. IB 1:5000.

Secondary antibodies (immunofluorescence)
1. Anti-Mouse IgG (H+L) Highly Cross-Adsorbed Secondary Antibody, Alexa Fluor™ 594, Invitrogen. IF 1:200. Cat# A-11032.

2. Anti-Rabbit IgG (H+L) Highly Cross-Adsorbed Secondary Antibody, Alexa Fluor™ 647, Invitrogen. IF 1:200. Cat# A-21245.

Validation

All primary antibodies used in this study are have been validated by manufacturer and/or in literature.
Furthermore, they were successfully utilized in wild type samples before they were employed in our experimental settings.

1. Anti-NNT, Invitrogen, Cat# 459170, validated by manufacturer (https://www.thermofisher.com/order/genome-database/dataSheetPdf?producttype=antibody&productsubtype=antibody_primary&productId=459170&version=214) and in untransfected WT HEK293 cells in this manuscript (Fig. 2e,f).

2. Anti-NNT(G-8), Santa Cruz, Cat# sc-390215, validated in Usami, M., et al. 2018, Genetic differences in C57BL/6 mouse substrains affect kidney crystal deposition, Urolithiasis 46: 515-522 and Francisco, A., et al. 2020, Mitochondrial NAD(P)+ transhydrogenase is unevenly distributed in different brain regions, and its loss causes depressive-like behavior and motor dysfunction in mice, Neuroscience. This antibody was also validated in this manuscript (Fig. 2g).

3. Anti NNT(B3)-HRP, Santa Cruz, Cat# sc-390236 HRP, validated in Rao, K.N.S., et al. 2020. Nicotinamide nucleotide transhydrogenase (NNT)regulates mitochondrial Ros and endothelial dysfunction in response to angiotensin II, Redox Biol. 36: 101650. This antibody was also validated in this manuscript (Fig. 2h).

4. Anti-PC1(C-term), Kerafast, Cat# EJH002, validated in Yu, S., et al. 2007, Essential role of cleavage of Polycystin-1 at G protein coupled receptor proteolytic site for kidney tubular structure, Proc Natl Acad Sci U S A, Nov 20;104(47):18688-93.

5. Anti-HA-Peroxidase (3F10), Roche, Cat# 12013819001, validated in Sapmaz, A. et al. 2019, USP32 regulates late endosomal transport and recycling through deubiquitylation of Rab7. Nat Commun, Mar 29;10(1):1454 and Buschauer, R., et al. 2020 The Ccr4-Not complex monitors the translating ribosome for codon optimality, Science, Apr 17;368(6488) as well as other publications.

6. Anti-HA-680, Thermo Fischer Scientific, Cat# 26183-D680, validated in McKenna, MJ., et al. 2020, The endoplasmic reticulum P5AATPase is a transmembrane helix dislocase, Science, Sep 25;369(6511):eabc5809 and in Zhang, Z., et al. 2013, Valproic acid causes proteasomal degradation of DICER and influences miRNA expression, PLoS One, Dec 17;8(12):e82895.

7. Anti-ki67, Vector Laboratories, Cat# VP-RM04, validated in Yan, P., et al. 2020, Genome-wide R-loop Landscapes during Cell Differentiation and Reprogramming, Cell reports , 32 (1) , 107870 and Lavado, A., et al. 2018, The Hippo Pathway Prevents YAP/TAZ Driven Hypertranscription and Controls Neural Progenitor Number, Developmental cell , 47 (5) , 576-591.e8 as well as other publications.

8. Anti- α5 subunit Na+K+ ATPase, Developmental Studies Hybridoma Bank, Cat# a5, validated initially in Lebovitz, RM., et al. 1989,Molecular characterization and expression of the (Na+ + K+)-ATPase alpha-subunit in Drosophila melanogaster, EMBO J, Jan;8(1):193-202 and later in Sakamori, R., et al. 2012, Cdc42 and Rab8a are critical for intestinal stem cell division, survival, and differentiation in mice, J Clin Invest,Mar;122(3):1052-65 as well as other publications.

9. Anti-actin, Sigma, Cat# A2228, validated in Ricciardi, S., et al. 2018, The Translational Machinery of Human CD4+ T Cells Is Poised for Activation and Controls the Switch from Quiescence to Metabolic Remodeling, Cell Metab, Dec 4;28(6):895-906.e5 and Allen, EH.,et al. 2016, Keratin 12 missense mutation induces the unfolded protein response and apoptosis in Meesmann epithelial corneal dystrophy, Hum Mol Genet, Mar 15;25(6):1176-91 as well as other publications.

10. Anti-TOMM20, Novus Biologicals, Cat# NBP1-81556, validated in Cassina, L., et al. 2020, Increased mitochondrial fragmentation in polycystic kidney disease acts as a modifier of disease progression, FASEB J, May;34(5):6493-6507 and Sala, D., et al. 2019, The Stat3-Fam3a axis promotes muscle stem cell myogenic lineage progression by inducing mitochondrial respiration, Nat Commun, Apr 17;10(1):1796.

11. Anti-Total OXPHOS Cocktail, Abcam, Cat# MS604-300, validated in Franczyk, MP., et al. 2021, Importance of Adipose Tissue NAD+ Biology in Regulating Metabolic Flexibility, Endocrinology, Mar 1;162(3):bqab006 as well as other publications. This antibody was also validated in this manuscript by rat heart tissue lysate (cat#110341, abcam) immunoblotting depicted in Fig. 5f.

12. Anti-VDAC-HRP, Santa Cruz, Cat# sc-390996 HRP, validated in Wei, L., et al. 2013. Oroxylin A induces dissociation of hexokinase II from the mitochondria and inhibits glycolysis by SIRT3-mediated deacetylation of cyclophilin D in breast carcinoma. Cell Death Dis. 4: e601 and Chen, Y., et al. 2015. Critical role of the neonatal Fc Receptor (FcRn) in the pathogenic action of antimitochondrial autoantibodies synergizing with anti-desmoglein autoantibodies in pemphigus vulgaris. J. Biol. Chem. 290: 23826-23837 as well as other publications.

13. Anti-aquaporin-2 (C-17), Santa Cruz, Cat# sc-9882, validated in O'Toole, J.F., et al. 2010. Individuals with mutations in XPNPEP3, which encodes a mitochondrial protein, develop a nephronophthisis-like nephropathy. J. Clin. Invest. 120: 791-802 as well as other publications.

14. Anti-NKCC2 - validated in Lytle, C., Xu, J.C., Biemesderfer, D. & Forbush, B., 3rd. Distribution and diversity of Na-K-Cl cotransport proteins: a study with monoclonal antibodies. Am J Physiol 269, C1496-505 (1995) as well as other publications.

15. Anti-megalin - validated in Zou, Z., Chung, B., Nguyen, T., Mentone, S., Thomson, B. & Biemesderfer, D. Linking receptor-mediated endocytosis and cell signaling: evidence for regulated intramembrane proteolysis of megalin in proximal tubule. J Biol Chem 279, 34302-10 (2004) as well as other publications.

Eukaryotic cell lines

Policy information about cell lines and Sex and Gender in Research

Cell line source(s)

HEK293 (ATCC, cat# CRL-1573); Pkd1-/- mTERT cells (generated for this manuscript).

Authentication	HEK293 cells were authenticated by ATCC through STR profiling, karyotyping and vitronectin expression.<br><br>Pkd1-/- mTERT cells were authenticated by immunofluorescence, western-blotting and quantitative PCR.
Mycoplasma contamination	No mycoplasma contamination - testing for mycoplasma performed with PCR-based Venor GeM Mycoplasma Detection Kit (catalog#MP0025, Sigma).
Commonly misidentified lines<br>(See ICLAC register)	No commonly misidentified cell lines were used in this study.

Animals and other research organisms

Policy information about studies involving animals; ARRIVE guidelines recommended for reporting animal research, and Sex and Gender in Research

Laboratory animals	Pkd1fl/fl;Pax8rtTA; TetO-Cre (+/- CTT) mice were generated on two distinct backgrounds by breeding in either C57BL/6J (stock no: 000664, Jackson Laboratories) or C57BL/6N (stock no:005304, Jackson Laboratories) strains.<br><br>The Pkd1fl/fl;Pkhd1-Cre model, originally generated on the C57BL/6J background, was crossed with 2HA-PC1-CTT; Pkd1 fl/fl; Pax8rtTA;TetO-Cre mice on the C57BL/6N background to produce an F1 progeny of Pkd1 fl/fl; Pkhd1-Cre (+/- CTT). F1 x F1 crossing generated "NN" and "JJ" Pkd1 fl/fl; Pkhd1-Cre (+/- CTT) F2 mice.<br><br>Previously characterized Pkd1F/H-BAC mice generated on a mixed strain background were used in this study (Fedeles, S.V., Tian, X., Gallagher, A.R., Mitobe, M., Nishio, S., Lee, S.H. et al. A genetic interaction network of five genes for human polycystic kidney and liver diseases defines polycystin-1 as the central determinant of cyst formation. Nat Genet 43, 639-47 2011).<br><br>Animals were maintained at a 12:12 light:dark cycle, with 30-70% humidity and a 20-26oC temperature. Male and female mice were used in all experiments. We ensured that sexually mature adult mice presented similar sex distribution across all experimental groups, as described in figure legends. In each experiment, animals were age-matched and sacrificed at either p14 (Pkd1 fl/fl; Pkhd1-Cre+/-CTT), 10 weeks (Pkd1fl/fl;Pax8rtTA;TetO-Cre+/-CTT and Pkd1F/H-BAC), 16 weeks (Pkd1fl/fl;Pax8rtTA;TetO-Cre+/-CTT)<br><br>Cre-negative littermates served as healthy WT controls.
Wild animals	Wild animals were not used in this study.
Reporting on sex	Sex distribution in sexually mature mice was similar across groups (specific descriptions in figure legends).
Field-collected samples	Field-collected animals were not used in this study.
Ethics oversight	All animals were used in accordance with humane, scientific and ethical principles and in compliance with regulations approved by Yale Animal Resources Center and Institutional Animal Care and Use Committee (IACUC). All mouse experiments were conducted in accordance with IACUC guidelines and procedures (protocol # 2019-20088).

Note that full information on the approval of the study protocol must also be provided in the manuscript.
